# Inhibition of Neutrophil Functions and Antibacterial Effects of Tarragon (*Artemisia dracunculus* L.) Infusion—Phytochemical Characterization

**DOI:** 10.3389/fphar.2020.00947

**Published:** 2020-08-13

**Authors:** Magdalena Majdan, Anna K. Kiss, Rafał Hałasa, Sebastian Granica, Ewa Osińska, Monika E. Czerwińska

**Affiliations:** ^1^Department of Pharmacognosy and Molecular Basis of Phytotherapy, Faculty of Pharmacy, Medical University of Warsaw, Warsaw, Poland; ^2^Department of Bromatology, Faculty of Pharmacy, Medical University of Warsaw, Warsaw, Poland; ^3^Department of Pharmaceutical Microbiology, Faculty of Pharmacy, Medical University of Gdańsk, Gdańsk, Poland; ^4^Department of Vegetable and Medicinal Plants, Institute of Horticulture Sciences, Warsaw University of Life Sciences—SGGW, Warsaw, Poland

**Keywords:** anti-inflammatory activity, antimicrobial activity, cytotoxicity, interleukin 8, reactive oxygen species, tumour necrosis factor, UHPLC-DAD-MS/MS

## Abstract

The aim of the study was to characterize phytochemicals in an infusion of the aerial parts of tarragon *(Artemisia dracunculus* L.) using ultra-high-performance liquid chromatography diode array detector electrospray ionisation tandem mass spectrometry UHPLC‐DAD‐ESI‐MS/MS method, as well as an evaluation of its effects on mediators of the inflammation in an *in vitro* model of human neutrophils, and antimicrobial activity on selected pathogens. Flavonoids and caffeoylquinic acids were the main phenolic components of the extract of tarragon’s aerial parts. The infusion was able to inhibit reactive oxygen species (ROS), interleukin 8 (IL-8), and tumour necrosis factor α (TNF-*α*) production. The antimicrobial assay was performed with the use of nine strains of bacteria, both Gram-negative and Gram-positive. Three human pathogens, *Staphylococcus aureus* ATCC6538, *Staphylococcus epidermidis* ATCC14990, and *Staphylococcus aureus* MRSA (methicyllin-resistant *Staphylococcus aureus*) ATCC43300, proved to be the most sensitive to tarragon infusion. Our study demonstrated the antiinflammatory and antimicrobial properties of tarragon *(Artemisia dracunculus* L.), meaning the common spice may be a prospective source of health-promoting constituents.

## Introduction

The popularity of spices as ingredients in food for the prevention and treatment of disease has increased in recent years. Tarragon (*Artemisia dracunculus* L.), also known as estragon, dragon wormwood, false tarragon, or dragon’s wort, is a species of a perennial herb of the *Asteraceae* family. The species name *dracunculus* is associated with the shape of the leaves, which is reminiscent of a dragon’s tongue (Aglarova et al., 2008). Informal names for distinguishing the main variety include French tarragon and Russian tarragon. While French tarragon is well-described in the recognized Western scientific literature, considerable information on Russian tarragon is covered in only a few publications (Govorko et al., 2007; Eisenman et al., 2011; Obolskiy et al., 2011). French tarragon is often used as a culinary herb, whereas Russian tarragon is bitter and more often used for medicinal preparations.

Tarragon comes from eastern and central Europe, southern Russia, and western Asia (Raghavan, 2006; Obolskiy et al., 2011). In the wild, tarragon grows on alkaline soils, near birch groves, near rivers and old fallow land, in steppe areas, and in the area of hills and in mountains. Tarragon grows well in any soil and under any temperature and lighting and tolerates spring and autumn frosts. Tarragon is most often propagated by seeds, but also by division of plants, as well as cuttings and root slips (Aglarova et al., 2008). *A. dracunculus* is cultivated for use of the leaves as an aromatic culinary herb and for medicinal purposes. The herb has a long history of use as a carminative, for stimulating the appetite, and detoxification of the liver (Obolskiy et al., 2011). The plant helps to alleviate the pain associated with dental diseases and also acts as an anti-inflammatory agent, and may also be useful in the treatment of microbial infections (Raghavan, 2006; Obolskiy et al., 2011; Eidi et al., 2016). Tarragon has also been used in the management of dysregulalated glucose metabolism, including hyperglycaemia, diabetes, and related metabolic syndromes (Obolskiy et al., 2011). *A. dracunculus* herb preparation has been applied widely for the treatment of skin wounds, irritations, allergic rashes, and dermatitis. A preliminary phytochemical study of *A. dracunculus* revealed the presence of essential oils (methyl eugenol, estragol, elemicin, terpinolene, and others), flavonoids (quercetin, luteolin, patuletin, kaemferol, isorhamnetin, naringenin, pinocembrin, and estragonoside and their glycosides), phenolic acids (cichoric acid, hydroxybenzoic acid, chlorogenic acid, caffeic acid, 5-*O*-caffeoylquinic acid, 4,5-di-*O*-caffeoylquinic acid, and others), coumarins (herniarin, coumarin, esculetin, esculin, capillarin, 8-hydroxycapillarin, artemidin, 8-hydroxyartemidin, artemidinol, and others) and alkamides (pellitorin, neopellitorin A, and neopellitorin B) (Sayyah et al., 2004; Logendra et al., 2006; Govorko et al., 2007; Aglarova et al., 2008; Eisenman et al., 2011; Miron et al., 2011; Obolskiy et al., 2011; Lin and Harnly, 2012). The chemical composition of *A. dracunculus* was investigated using specific reactions, UV/Vis spectroscopy, nuclear magnetic resonance spectroscopy NMR, chromatographic techniques (gas chromatography GC, high performance liquid chromatography HPLC), and mass spectrometry MS.

The aim of this work was to evaluate an infusion of Russian tarragon as a potential food product for the prevention and treatment of inflammation and bacterial infections. We investigated the effects of infusion on proinflammatory functions of human neutrophils, such as ROS production and IL-8 and TNF-*α* release. The anti-microbial activity was also checked using several pathogens. The comprehensive analysis of infusion *A. dracunculus’* aerial parts (ADI) in our studies has never been performed using the UHPLC‐DAD‐ESI‐MS/MS method.

## Materials and Methods

### Chemicals and General Experimental Procedures

Fetal bovine serum (FBS), f-MLP (N-Formylmethionyl-leucyl-phenylalanine), luminol, HEPES solution, RPMI 1640 medium, and L-glutamine were purchased from Sigma-Aldrich Chemie GmbH (Steinheim, Germany). Phosphate-buffered saline (PBS Ca^2+^-free) and penicillin-streptomycin were purchased from Gibco (Grand Island, USA). Lipopolysaccharide (LPS from *Escherichia coli* 0111:B4) was purchased from Merck (Kenilworth, USA). Pancoll human was obtained from PAN Biotech (Aidenbach, Germany). Human Quantikine ELISA Kits and propidium iodide were purchased from BD Biosciences (San Jose, USA). Dexamethasone (DEX) and quercetin (QU) (> 95% UHPLC purity) were purchased from Sigma-Aldrich GmbH (Steinheim, Germany). Acetonitrile, methanol, and formic acid for UHPLC were purchased from Merck (Darmstadt, Germany). Water for UHPLC was purified with a Millipore Simplicity System (Bedford, USA). All solvents used for chromatography were of HPLC grade. Absorbance and luminescence were measured using a BioTek microplate reader (Highland Park, USA). Flow cytometry was performed using a BD FACSCalibur apparatus (BD Biosciences, San Jose, USA). Brain-heart infusion broth (BHI) and Mueller-Hinton broth (MH cation-adjusted) were purchased from Becton Dickinson (Franklin Lakes, USA).

### Plant Material

The aerial parts of Russian tarragon were collected in August 2014 from the experimental field of the Department of Vegetables and Medicinal Plants in Wilanów, Warsaw, Mazovian district, Poland (21°0099109 E 52°162209 N). The plant material was authenticated by Prof. Ewa Osińska (Warsaw University of Life Sciences, Poland) according to a guidebook (Rutkowski, 2006). Voucher specimens no. 121 were deposited at the herbarium of the Department of Vegetable and Medicinal Plants, Warsaw University of Life Sciences.

### Preparation of ADI and Its Phytochemical Characterization by UHPLC-DAD-MS/MS Method

A 3 g portion of air-dried plant material was poured into boiling water (250 mL), covered, and allowed to stand for 15 min (3×) in a tea infuser (Ambition, Warsaw, Poland). Extracts were then filtered and lyophilized (lyophilizer Telstar Cryodos 50, Telstar International, S.L., Terrassa, Spain), resulting in the following yields: sample 1 – 1.32 g, sample 2 – 0.98 g, sample 3 – 1.24 g. UHPLC-DAD-MS analysis was conducted using a Dionex Ultimate 3000RS system coupled with an Amazon SL ion trap mass spectrometer (Bruker Daltonics, Bremen, Germany). The ion trap AmazonSL mass spectrometer was equipped with an ESI interface. The eluate was introduced into the ESI interface of the mass spectrometer without splitting. The parameters for the ESI source were as follows: nebulizer pressure 40 psi; dry gas flow 9 L/min; dry temperature 300°C; and capillary voltage 4.5 kV. Analysis was carried out using scanning from *m/z* 70 to 2200. Mass spectra were recorded in positive- and negative-ion modes. A sample of the crude plant extract dissolved in methanol (20 mg/mL) was filtered through a 0.45 μm syringe filter and subjected to UHPLC-DAD-MS analysis. The separation was carried out on a Kinetex XB-C_18_ (150 mm × 3.0 mm × 2.6 μm, Phenomenex, Torrance, CA, USA) column maintained at 25°C. The mobile phases were 0.1% HCOOH in water (A) and 0.1% HCOOH in acetonitrile (B), and elution was conducted with the following gradient: 0 min – 0% B; 60 min – 26% B; and 80 min, 90% B. The flow rate was 0.4 mL/min, and the injection volume was 3 μL of the prepared extract. The UV−Vis spectra of the detected compounds were recorded over the 190 - 450 nm range. The chromatogram was recorded at 280 nm and 350 nm. Compounds were characterized based on the maxima observed in their UV−Vis spectra and on their MS spectra.

### Isolation of Human Neutrophils

The buffy coats were prepared from peripheral venous blood collected from healthy human donors (< 35 years old) at the Warsaw Blood Donation Centre. Donors were confirmed to be healthy and all tests carried out showed values within a normal range. Donors did not smoke or take any medications. The study conformed to the principles of the Declaration of Helsinki. Neutrophils were isolated using a standard method by dextran sedimentation and centrifugation in a Pancoll gradient (Böyum, 1968). After isolation, cells were suspended in (Ca^2+^)-free HBSS or RPMI 1640 culture medium.

### Evaluation of ADI Cytotoxicity

Cytotoxicity was determined by flow cytometry using propidium iodide (PI) staining. After 24 h of incubation in the standard conditions (37°C, 5% CO_2_) with extracts or standards used as positive controls in tests, the neutrophils were harvested and centrifuged (1500 RPM; 10 min; 4°C), washed once with cold PBS, and re-suspended in 500 µL of PBS. Five microliters of PI (50 µg/mL) solution was added to the cell suspensions. After 15 min of incubation at room temperature, cells were analyzed by flow cytometry, and 10000 events were recorded per sample. Cells that displayed high permeability to PI were expressed as a percentage of PI (+) cells. Triton X was used as positive control.

### Measurement of ROS Production

The ROS production by f-MLP-stimulated neutrophils was determined using luminol-dependent chemiluminescence. ADI were tested at concentrations of 12.5, 25, 50, and 100 μg/mL. Following isolation, cells were suspended in 70 μL (Ca^2+^)-free HBSS. Cell suspension (3.0×10^5^/mL) was incubated with 50 μL of the samples with tested concentrations of extract and 50 μL of luminol (100 μM). ROS production was initiated by the addition of 30 μL of f-MLP (0.1 μg/mL). Changes in the chemiluminescence were measured over a 40 min period at intervals of 2 min in a microplate reader (BioTek, Synergy 4) at 37°C. Background chemiluminescence produced by non-stimulated cells was also checked. The tested extract did not interfere with the chemiluminescence signal. As a positive control, quercetin was used at a concentration of 20 μM. The percentage of ROS production was calculated in comparison to the control without investigated tarragon water extract.

### IL-8 and TNF-*α* Release

Neutrophils (2 × 10^6^ cells/mL) were cultured in RPMI 1640 medium with 10% FBS, 10 mM HEPES, and 2 mM L-glutamine for 24 h at 37°C with 5% CO_2_ in the absence or presence of extract at final concentrations of 12.5, 25, 50, and 100 μg/mL (96-well plates, 1 mL per well) 1 h before stimulation LPS (100 ng/mL). After 24 h, plates were centrifuged (2000 RPM; 10 min; 4°C) and supernatants were collected. The release of cytokines by stimulated neutrophils was evaluated by enzyme-linked immunosorbent (ELISA) tests following the manufacturer’s instructions (BD Biosciences, San Jose, CA, USA or R&D Systems, Minneapolis, MN, USA). Dexamethasone at concentrations of 12.5, 25, and 50 μM and quercetin at concentration of 50 μM were used as a positive control for the release of IL-8 and TNF-*α*.

### Statistical Analysis

The results were expressed as the mean ± SEM of three independent experiments performed in triplicate. The statistical significance of differences between means and control was determined by ANOVA with Tukey’s *post hoc* test. P values below 0.05 were considered statistically significant with Statistica 10 software (Statsoft, Poland).

### Antimicrobial Assay

The antibacterial activity was assessed against Gram-positive (*Staphylococcus aureus* ATCC6538, *Staphylococcus aureus* MRSA ATCC43300, *Staphylococcus epidermidis* ATCC14990, *Enterococcus hirae* ATCC10541, and *Corynebacterium diphtheriae*) and Gram-negative (*Escherichia coli* ATCC8739, *Klebsiella pneumoniae* ATCC13883, *Proteus vulgaris* NCTC4635, and *Helicobacter pylori* ATCC43504) reference strains. All bacteria were obtained from the Department of Pharmaceutical Microbiology, Medical University of Gdańsk’s collection. Brain-heart infusion broth (BHI, Becton Dickinson) was used for breeding of *E. hirae* and supplemented with 10% bovine serum for *C. diphtheriae* (grown in aerobic conditions at 37°C for 48 h) (Lekogo et al., 2010). Mueller-Hinton broth (MH cation-adjusted, Becton Dickinson) was used for: *S. aureus, E. coli, K. pneumoniae*, and *P. vulgaris* (grown in aerobic conditions at 37°C for 48 h). BHI (Becton Dickinson) supplemented with 5% horse serum was used for *H. pylori* (grown in microaerophilic conditions, GENbag microaer, BioMerieux at 37°C for 72 to 96 h). The antibacterial assay was performed according to a previously established method (Kula et al., 2013). Dry extract (1 g) was dissolved in the sterile distilled water (2 mL). The final concentrations of the extracts used for the antimicrobial activity ranged from 0.004 to 94.000 mg/mL. The lowest concentration at which no visible growth was taken as the MIC (minimal inhibitory concentration).

## Results

### Phytochemical UHPLC-DAD-MS/MS Characterization of ADI

**Figure 1:**
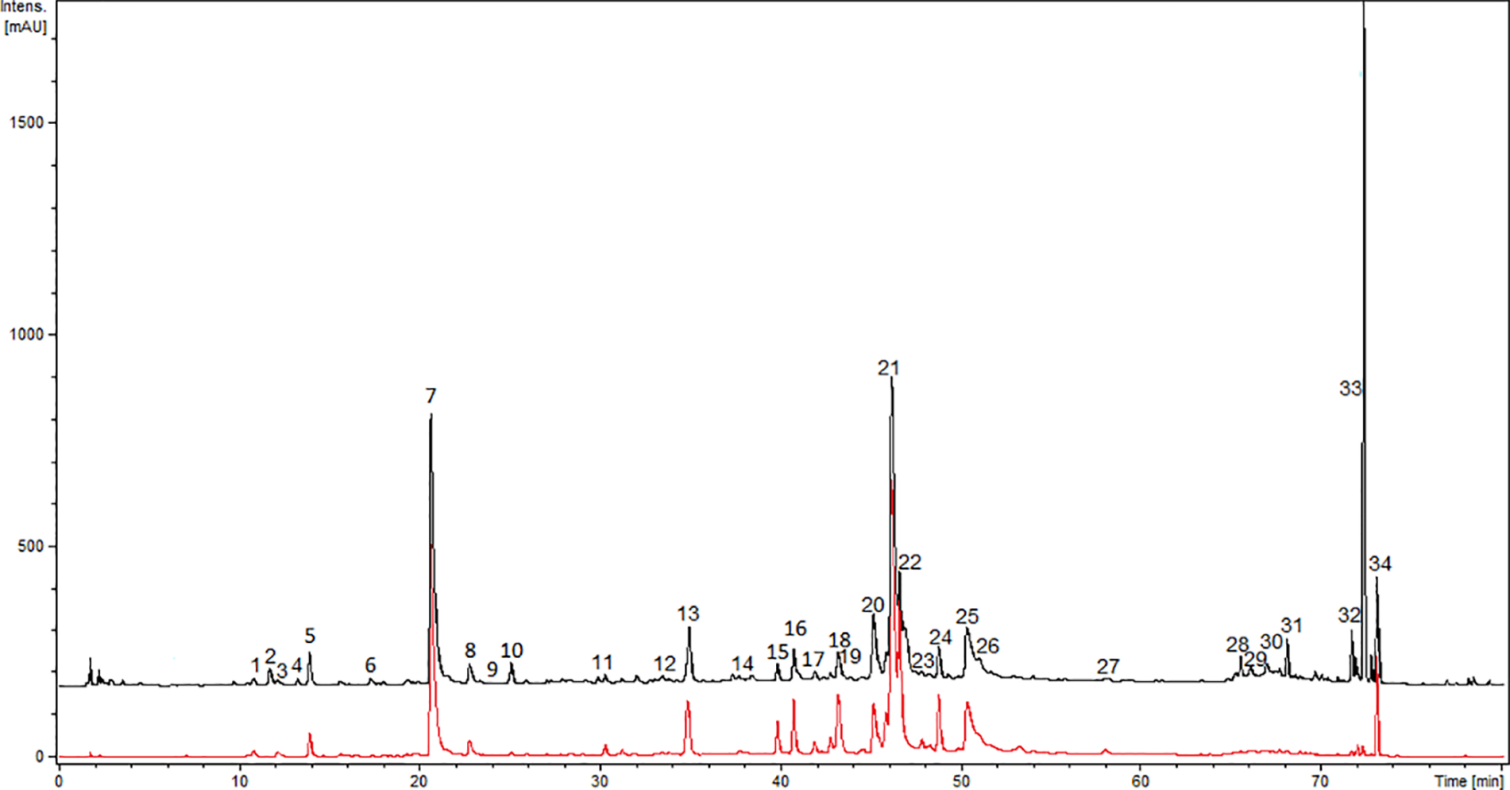
UHPLC-DAD-ESI-MS/MS chromatograms of ADI recorded at 280 nm (black line) and 350 nm (red line).

Further, the comprehensive analysis of ADI was performed with the UHPLC-DAD-MS/MS method. Chromatograms (280 nm and 350 nm) of ADI are depicted in **Figure 1**. We were able to identify or partly identify 34 compounds from different chemical groups (**Table 1**). The chemical profile was found to be dominated by flavonoids (**11, 14, 15, 16, 17, 18, 19, 21, 23, 25, 29, 31, 32, 34**) and caffeoylquinic acids (**1, 5, 7, 8, 20, 22, 24, 26**). Moreover, other phenolic acid derivatives were also identified in the analysed sample. The retention times (t_r_), wavelength of maximum absorbance (λmax), pseudomolecular ions ([M+H]^+^/[M−H]^−^), and major fragment ions are listed in **Table 1**. All eight of the caffeoylquinic acids (caffeoylquinic acids and di-*O*-caffeoylquinic acids), fourteen of the flavonoids (quercetin, isorhamnetin, syringetin, apigenin, patuletin derivatives, davidigenin, sakuranetin, 2′,4′-dihydroxy-4-methoxydihydrochalcone), four phenolic acid derivatives (protocatechuic acid, caffeoyl hexaric acid, syringic acid, ferulic acid) and one coumarin (6-demethoxycapillarisin) were positively identified by the comparison of their chromatographic data with the literature (Logendra et al., 2006; Eisenman et al., 2011; Miron et al., 2011; Barros et al., 2012; Lin and Harnly, 2012; Gu et al., 2012; Olennikov et al., 2018; Mekky et al., 2019; Olennikov et al., 2019; Kiss et al., 2020). The main pseudomolecular ion obtained for compounds **1** (t_r_ =11.7 min), **5** (tr =14 min), **7** (t_r_ =20.9 min), and **8** (t_r_ =22.8 min) was at *m/z* 353 [M–H]^-^. In addition, the presence of an intensive signal at *m*/*z* 191 in MS/MS spectra, which is indicative of quinic acid moiety, was established (El-Askarya et al., 2019). The compounds were further assigned based on the fragmentation patterns and their elution order as mono-*O*-caffeoylquinic acids (Eisenman et al., 2011; Miron et al., 2011; Gu et al., 2012; Lin and Harnly, 2012; Olennikov et al., 2018; Olennikov et al., 2019; Kiss et al., 2020). Four compounds **20** (t_r_ =45 min), **22** (t_r_ = 46.1 min), **24** (t_r_ = 47.8 min), and **26** (t_r_ = 50.3 min) with UV and MS spectra ([M–H]^−^ ion with *m*/*z* 515 and fragment ions with *m*/*z* 353 and 191) were assigned the structure di-*O*-caffeoylquinic acids (1,4-di-*O*-CQA, 3,4-di-*O*-CQA, 3,5-di-*O*-CQA, 1,5-di-*O*-CQA or 4,5-di-*O*-CQA) (Eisenman et al., 2011; Miron et al., 2011; Gu et al., 2012; Lin and Harnly, 2012; Olennikov et al., 2018; Olennikov et al., 2019). Further, compounds **2** (t_r_ = 12 min), **3** (t_r_ = 12.2 min), **4** (t_r_ = 13.4 min), **10** (t_r_ = 25 min) and **13** (t_r_ = 34.9 min) gave pseudomolecular ions *m*/*z* [M−H]^–^477, 371, 359, 355, respectively, and were tentatively identified as protocatechuic acid dihexoside, caffeoyl hexaric acid, syringic acid hexoside, and ferulic acid hexoside, respectively (Barros et al., 2012; Mekky et al., 2019; Kiss et al., 2020). Flavonoid glycosides were assigned as derivatives of isorhamnetin (Miron et al., 2011; Lin and Harnly, 2012), apigenin (Lin and Harnly, 2012), patuletin (Vienne et al., 1989; Lin and Harnly, 2012), and quercetin (Obolskiy et al., 2011; Lin and Harnly, 2012). The large group of flavonoids, including flavonols, flavones, flavanones, dihydrochalcones, *O*- and *C*-glycosides, was detected in ADI (**11**, **14**, **15**, **16**, **17**, **18**, **19**, **21, 23, 25, 29, 31, 32, 34**). Compound **11** (t_r_ = 30.2 min) was tentatively identified as vicenin-2 (apigenin 6,8-di-*C*-glucoside) by comparison with the literature (Lin and Harnly, 2012; Olennikov et al., 2018) of its UV spectrum (270, 333 nm) and mass spectrometric data ([M+H]^+^/[M−H]^–^ pseudomolecular ions with 595/593 *m/z*, [M+H]^+^ 595 *m/z* →457, 577 *m/z*). In fact, it was previously identified among others in the *A. herba- alba* Asso aerial part by Saleh et al. (Saleh et al., 1987). Compound **15** (t_r_ = 39.8 min) was characterized by UV maxima at 256 nm and 355 nm, typical for flavonol glycosides (de Rijke et al., 2006). The pseudomolecular ion was at *m/z* 609 [M−H]^–^ and gave a main fragment ion at *m/z* 301 [M−H]^–^ attributed to quercetin, and the loss of 308 amu was due to a cleavage of hexosylrhamnoside sugar moiety. The compound **15** was then identiﬁed as quercetin-3-*O*-rhamnosylhexoside, a characteristic flavonoid for *Artemisia* sp. (Lin and Harnly, 2012; Olennikov et al., 2018). Isoquercitrin/hiperoside were identified as compound **14 (**Olennikov et al., 2018**)** and compound **29** was tentatively assigned as free quercetin (Obolskiy et al., 2011; Gu et al., 2012; Lin and Harnly, 2012; Olennikov et al., 2018). Compound **18** exhibited the pseudomolecular ion was at *m/z* [M+H]^+^/[M−H]^–^ 697/695 and gave a main fragment ion at *m/z* 303/301 attributed to quercetin (Obolskiy et al., 2011; Gu et al., 2012; Lin and Harnly, 2012; Olennikov et al., 2018). Furthermore, compounds **16** (t_r_ = 40.8 min), **17** (t_r_ =41.9 min), **19** (t_r_ = 43.3 min), and **25** (t_r_ = 48.7 min) were characterized by UV maxima at 266 nm and 335 nm, typical for flavonoids (de Rijke et al., 2006) and exhibited the main ion at *m/z* [M+H]^+^/[M−H]^–^ 333/331 attributed to patuletin, which seemed to occur only in the Russian cultivar (Vienne et al., 1989; Bohm and Stuessy, 2001). Compound **16** exhibited pseudomolecular ion [M+H]^+^/[M−H]^–^at *m/z* 641/639 and fragment ion at *m*/*z* 333/331, and was attributed to patuletin rhamnosylhexoside. The ions at *m*/*z* 495 corresponded to the sequential losses of rhamnosyl residue (146 amu). The compound was then tentatively assigned as patuletin 3-*O*-robinobioside (Lin and Harnly, 2012). Since compound **17** (M+H]^+^/[M−H]^–^ at *m/z* 495/493) contained one glucoside residue (162 amu), it was thus tentatively identified as patuletin hexoside. Compounds **19** and **25** have been previously reported in tarragon (Lin and Harnly, 2012). Flavonoid glycosides, such as patuletin 3-*O*-malonylrobinobioside and patuletin 3-*O*-malonylrhamnosylhexoside, have been identified. Peak **19** exhibited pseudomolecular ions at *m/z* 727/725 [M+H]^+^/[M−H]^–^ and fragments ions at *m/z* [M−H−44] ^–^ as well as *m/z* [M+H−162]^+^ corresponding to the cleavage of malonyl and hexose moieties, respectively. The pseudomolecular ion of compound 23 was at *m/z* 655 [M+H]^+^ and gave a main fragment ions at *m/z* [M+H]^+^ 347, attributed to syringetin. The loss of 308 amu is due to a cleavage of hexosylrhamnoside sugar moiety and ion at *m*/*z* 509 [M+H]^+^ resulting from the concomitant losses of a rhamnose (146 amu). Compound **23** (t_r_ = 46.8 min) was tentatively assigned as syringetin 3-*O*-rhamnosylhexoside, identified in *Artemisia dracunculus* (Lin and Harnly, 2012). Additionally, compound **25** was identified as patuletin 3-*O*-malonylrhamnosylhexoside, with pseudomolecular ions at *m/z* 741/739 [M+H]^+^/[M−H]^–^. Compounds **30** (t_r_ = 66.9 min) and **32** (t_r_ = 71.6 min) exhibited a pseudomolecular [M-H]^-^ ion at 285 *m/z*, indicating that compounds were 6-demethoxycapillarisin and sakuranetin, according to the elution (Logendra et al., 2006; Govorko et al., 2007; Eisenman et al., 2011). Compound **31** (t_r_ = 68.1), which gave a pseudomolecular ion [M–H]^−^ with *m/z* 257 in the negative ESI mode, was identified as davidigenin (Logendra et al., 2006; Eisenman et al., 2011). The analysed extract of *A. dracunculus* contained compound **34**, (t_r_ = 73.0 min), which gave pseudomolecular ions [M–H]^−^ at *m*/*z* = 271 in the negative ionization and were identified as 2′,4′-dihydroxy-4-methoxydihydrochalcone, described early by Eisenman et al., (2011), Govorko et al., (2007), and Logendra et al., (2006).

**Table 1:** Constituents annotated in ADI extract by UHPLC-DAD-ESI-MS.

Compounds	UV[nm]	Rt[min]	[M+H]^+^ (major and fragments *m/z*)	[M-H]^-^ (major and fragments *m/z*)	Identification	References
**1**.	215, 296, 325	11.7	355 (163)	353 (191)	caffeoylquinic acid (I)	(Eisenman et al., 2011; Gu et al., 2012; Lin and Harnly, 2012; Olennikov et al., 2018; Olennikov et al., 2019)
**2**.	216, 278	12.0		477 (315, 153)	protocatechuic acid dihexoside	
**3**.	212, 282,320	12.2		371 (209, 191)	caffeoyl hexaric acid	(Kiss et al., 2020)
**4**.	212, 260	13.4		359 (197)	syringic acid hexoside	(Barros et al., 2012)
**5**.	215, 286, 323	14.0	355 (163)	353 (191)	caffeoylquinic acid (II)	(Eisenman et al., 2011; Gu et al., 2012; Lin and Harnly, 2012; Olennikov et al., 2018; Olennikov et al., 2019)
**6**.	214	17.8	360 (163, 307), 325 (163,307)	387 (179, 285, 341)	unknown	
**7**.	217, 286, 324	20.9	355 (163)	353 (191)	caffeoylquinic acid (III)	(Eisenman et al., 2011; Miron et al., 2011; Gu et al., 2012; Lin and Harnly, 2012; Olennikov et al., 2018; Olennikov et al., 2019)
**8**.	216, 286, 324	22.8	355 (163)	353 (191, 179, 173)	caffeoylquinic acid (IV)	(Eisenman et al., 2011; Miron et al., 2011; Gu et al., 2012; Lin and Harnly, 2012; Olennikov et al., 2018; Olennikov et al., 2019)
**9**.	202	23.3	225	447 (401)	unknown	
**10**.	235, 300	25.0	374 [M+Na]^+^ (195, 177)	355 (193, 149)	ferulic acid hexoside	(Lin and Harnly, 2012; Mekky et al., 2019)
**11**.	270, 333	30.2	595 (457, 577)	593 (575, 503, 473, 383, 353)	vicenin - 2 apigenin 6,8-di-C-glucoside	(Gu et al., 2012; Lin and Harnly, 2012; Olennikov et al., 2018; Olennikov et al., 2019)
**12**.	217, 288, 324	34.1	544 (365)	525, (481, 433, 301)	unknown	
**13**.	207, 230, 319	34.9	–	355 (193, 149)	ferulic acid hexoside	(Lin and Harnly, 2012; Mekky et al., 2019)
**14**.	215, 280, 342	37.8	465	463 (301)	isoquercitrin/hiperoside	(Olennikov et al., 2018)
**15**.	215, 256, 355	39.8	611 (465, 303)	609 (301, 487)	quercetin-3-*O*-rhamnosylhexoside	(Lin and Harnly, 2012; Olennikov et al., 2018)
**16**.	210, 260, 336	40.8	641 (495, 333), 333 (318, 222)	639 (331)	patuletin rhamnosylhexoside	(Lin and Harnly, 2012)
**17**.	212, 262, 341	41.9	495	493	patuletin hexoside	(Lin and Harnly, 2012)
**18**.	201, 260, 347	42.8	697 (535, 303)	695 (651, 609, 301)	quercetin derivative	(Lin and Harnly, 2012; Olennikov et al., 2018)
**19**.	203, 255, 346	43.3	727 (565, 495, 333)	725 (681, 639, 331)	patuletin 3-*O*-malonylrobinobioside	(Lin and Harnly, 2012)
**20**.	216, 286, 325	45.0	517 (499, 355)	515 (353, 191, 179,173)	di-*O*-caffeoylquinic acid (I)	(Eisenman et al., 2011; Miron et al., 2011; Gu et al., 2012; Lin and Harnly, 2012; Olennikov et al., 2018; Olennikov et al., 2019)
**21**.	–	45.9	625 (479, 317)	623 (315)	isorhamnetin rhamnosylhexoside	(Lin and Harnly, 2012)
**22**.	216, 245, 292, 325	46.1	517	515 (353, 191, 179)	di-*O*-caffeoylquinic acids (II)	(Eisenman et al., 2011; Miron et al., 2011; Gu et al., 2012; Lin and Harnly, 2012; Olennikov et al., 2018; Olennikov et al., 2019)
**23**.	–	46.8	655 (509, 347)	653 (345, 330)	syringetin 3-*O*-rhamnosylhexoside	(Lin and Harnly, 2012)
**24**.	216, 325	47.8	517 (499)	515 (353, 191, 179, 173)	di-*O*-caffeoylquinic acids (III)	(Eisenman et al., 2011; Miron et al., 2011; Gu et al., 2012; Lin and Harnly, 2012; Olennikov et al., 2018; Olennikov et al., 2019)
**25**.	210, 255, 344	48.7	741 (579, 509, 347)	739 (695)	patuletin malonylrhamnosylhexoside	(Lin and Harnly, 2012)
**26**.	216, 293, 325	50.3	517 (499)	515 (353, 191, 179, 173)	di-*O*-caffeoylquinic (IV)	(Eisenman et al., 2011; Miron et al., 2011; Gu et al., 2012; Lin and Harnly, 2012; Olennikov et al., 2018; Olennikov et al., 2019)
**27**.	220, 319	58.0	–	471 (402, 309, 240)	unknown	
**28**.	216, 271, 312	65.5	581 (435, 419, 273)	579 (271)	unknown	
**29**.	–	66.0	303 (285)	301 (151)	quercetin	(Obolskiy et al., 2011; Gu et al., 2012; Lin and Harnly, 2012; Olennikov et al., 2018)
**30**.	220, 285	66.9	287 (269)	285 (267, 257, 241, 229)	6-demethoxycapillarisin	(Logendra et al., 2006; Eisenman et al., 2011; Govorko et al., 2007)
**31**.	216, 276, 315	68.1	259	257 (151)	davidigenin	(Logendra et al., 2006; Eisenman et al., 2011)
**32**.	220, 286	71.6	287 (241, 167, 147)	285 (269, 263, 175, 163,151)	sakuranetin	(Eisenman et al., 2011)
**33**.	196, 230, 263	72.3	–	299 (283, 191)	unknown	
**34**.	211, 228, 297, 335	73.0	–	271 (256, 150),	2′,4′-dihydroxy-4-methoxydihydrochalcone	(Logendra et al., 2006; Govorko et al., 2007; Eisenman et al., 2011)

### Effect of ADI on Pro-Inflammatory Functions of Stimulated Neutrophils and Cytotoxicity

**Figure 2:**
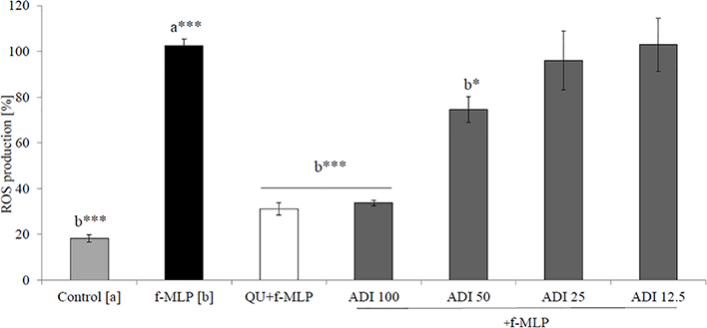
Effect of the ADI at concentrations of 12.5-100 µg/mL on ROS production by f-MLP-stimulated neutrophils [%], mean ± SEM. Positive control quercetin (QU) at a concentration of 20 µM. Statistical significance of differences was established by ANOVA with *post hoc* test, *p < 0.05 ***p < 0.001, a*** - statistical significance of differences between f-MLP stimulated control compared control, b - compared to the to the stimulated control f-MLP.

**Figure 3:**
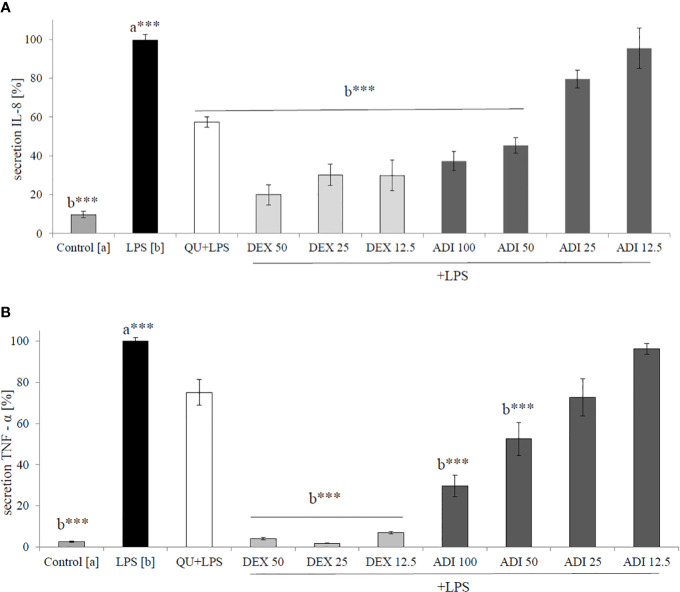
Effect of ADI at concentrations of 12.5 - 100 µg/mL on **(A)** IL-8 production and **(B)** TNF-*α* production by LPS-stimulated neutrophils [%], mean ± SEM. Positive controls quercetin (QU) at a concentration of 50 µM and dexamethason (DEX) at concentrations 50, 25, and 12.5 µM. Statistical significance of differences was established by ANOVA with *post hoc* test, ***p < 0.001, a*** - statistical significance of differences between LPS-stimulated control compared to control, b - compared to the stimulated control LPS.

**Figure 4:**
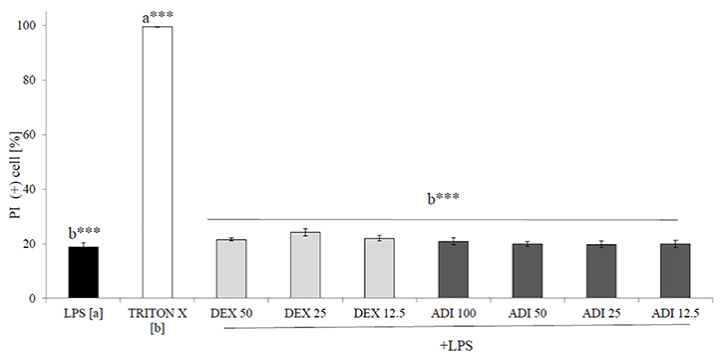
The cytotoxic effect of ADI at concentrations of 12.5 - 100 µg/mL on LPS-stimulated neutrophils after 24 h incubation [%], mean ± SEM. Statistical significance of differences was established by ANOVA with *post hoc* test, ***p<0.001, a*** - statistical significance of differences between LPS-stimulated control compared to control cells (Triton X), b - compared to control cells (Triton X).

Neutrophils, also known as polymorphonuclear cells (PMNs), after infiltration to the inflammation site, generate ROS. Stimulation by f-MLP (bacterial derived factor) results in degranulation and the significant release of ROS compared to the non-stimulated control (**Figure 2**). Incubation of stimulated neutrophils with ADI in the concentration range of 12.5 - 100 μg/mL turned out to inhibit the production of ROS (**Figure 2**). Quercetin at a concentration of 20 µM was used as a positive control in performed experiments. The most significant activity was noticed for ADI at a concentration of 100 µg/mL with the decrease of ROS production down to 33.6 ± 1.4% compared with the stimulated control for f-MLP (p < 0.001). Furthermore, LPS-stimulated neutrophils were used for the assessment of IL-8 and TNF-*α* release inhibition (**Figures 3A, B**). Tarragon (*Artemisia dracunculus* L.) infusion at concentrations of 50 and 100 µg/mL was able to inhibit the secretion of IL-8 by human neutrophils (45.4 ± 4.0% and 37.3 ± 4.8% compared to LPS stimulated control 99.7 ± 2.6%, p<0.001). At concentrations of 50 and 100 μg/mL, a significant decrease of the production of TNF-*α* from stimulated PMNs was also observed (52.5 ± 7.8% and 29.7 ± 5.2% compared to LPS stimulated control 99.9 *±* 1.8%, p<0.001). However, the observed effects for the TNF-*α* release were less relevant than for the positive control (dexamethasone at concentrations 12.5, 25, 50 µM, **Figure 3B**). ADI at lower concentrations of 12.5 and 25 μg/mL did not affect the release of IL-8 and TNF-*α* from LPS-stimulated neutrophils (no statistically significant differences were observed). The effect is probably connected with the presence of polyphenolic compounds such as caffeoylquinic acids and flavonoids in ADI. Tarragon infusion at concentrations of 12.5, 25, 50, and 100 μg/mL have shown no statistically significant reduction in cells’ membrane integrity in comparison to the control cells in a propidium iodide assay (**Figure 4**). At higher concentrations no differences between samples treated with ADI and LPS-stimulated cells were observed. A common anti-inflammatory drug used in this study – dexamethasone - at all tested concentrations did not influence the viability of cells compared to LPS control.

### Antibacterial Activity

The antimicrobial activity of ADI was evaluated against nine strains of human pathogenic bacteria compared to the ampicillin (**Table 2**). The extract was the most active against *Staphylococcus aureus* ATCC6538 (MIC 0.09 mg/mL). Similarly, the extract demonstrated some inhibitory effect against *Staphylococcus epidermidis* ATCC14990 (MIC 0.363 mg/mL) and *Staphylococcus aureus* MRSA ATCC43300 (MIC 2.35 mg/mL). ADI with an MIC value of 5.9 mg/mL was active against *Corynebacterium diphtheriae* – a bacterium causing different types of diphtheria affecting the respiratory tract and skin. Our results show a growth inhibiting effect of *A. dracunculus* extracts against *Helicobacter pylori* (MIC 11.75 mg/mL). The analysed extract showed weak activity towards strains *Enterococcus hirae* ATCC10541 (MIC 23.5 mg/mL) and *Klebsiella pneumoniae* ATCC13883 (47 mg/mL). The two insensitive strains proved to be *Proteus vulgaris* NCTC4635 and *Escherichia coli* ATCC8739.

**Table 2:** Antimicrobial activity of ADI and ampicillin [mg/mL].

	ADI[MIC]	Ampicylin[MIC]
*Staphylococcus aureus* ATCC6538	0.09	<0.00003
*Staphylococcus aureus* MRSA ATCC43300	2.35	0.00024
*Staphylococcus epidermidis* ATCC14990	0.363	0.000025
*Enterococcus hirae* ATCC10541	23.5	0.0001
*Corynebacterium diphtheria*	5.9	0.000025
*Escherichia coli* ATCC8739	>94	0.0001
*Klebsiella pneumoniae* ATCC13883	47	0.00195
*Proteus vulgaris* NCTC4635	94	0.0005
*Helicobacter pylori* ATCC43504	11.75	0.0032

## Discussion

Various genetic and environmental factors may influence the biosynthesis and chemical compositions of plant secondary metabolites (Deng et al., 2019). In the present study, the chemical composition of ADI was identified using UHPLC‐DAD‐ESI‐MS/MS analysis. The most important classes of biologically active substances in the aerial part of tarragon are flavonoids, phenolic acid derivatives, the essential oil, coumarins, and alkamides (Logendra et al., 2006; Govorko et al., 2007; Aglarova et al., 2008; Eisenman et al., 2011; Miron et al., 2011; Obolskiy et al., 2011; Lin and Harnly, 2012). Previous studies revealed that extract from tarragon contained characteristic coumarins, herniarin, esculetin, esculin, capillarin, 8-hydroxycapillarin, artemidin, 8-hydroxyartemidin, artemidinol, and others (Aglarova et al., 2008; Bhutia and Valant-Vetschera, 2008; Obolskiy et al., 2011). The other reports focused on chromatographic analysis of extracts from tarragon (Hoffmann and Herrmann, 1982; Aglarova et al., 2008; Miron et al., 2011; Obolskiy et al., 2011; Lin and Harnly, 2012) and showed that they contained flavonoids, including quercetin, apigenin, isorhamnetin, and their glycosides. Those results are in agreement with the current work, in which we identified similar flavonoids and phenolic acids derivatives (**Table 1**, **Figure 1**). The main flavonoids’ components of tarragon herb were the glycosides of patuletin. They have been reported by Bohm and Stuessy (Bohm and Stuessy, 2001) in *Asteraceae* plants. The rest of the detected components, by UHPLC‐DAD‐ESI‐MS/MS method, were identified as caffeoylquinic acids. Based on literature data (Logendra et al., 2006; Govorko et al., 2007; Eisenman et al., 2011; Miron et al., 2011; Obolskiy et al., 2011; Barros et al., 2012; Gu et al., 2012; Lin and Harnly, 2012; Olennikov et al., 2018; Olennikov et al., 2019; Kiss et al., 2020) we have determined the phenolic profile of *A. dracunculus* herb infusion included 34 compounds, predominantly caffeoylquinic acids. Caffeoylquinic acids are known powerful antioxidants and plant sources of caffeoylquinic acids, such as *A. dracunculus* extracts, which may provide antioxidant protection. A high caffeoylquinic acids content was previously found in other *Artemisia* plants, such as *A. annua* L. (El-Askarya et al., 2019) and *A. herba-alba* (Bourgou et al., 2017). *A. dracunculus* herb infusion can also be a good source of caffeoylquinic acids in the everyday diet. The use of fresh leaves or extracts from *A. dracunculus* is found in traditional medicine and has been partially supported by *in vitro* and *in vivo* studies (Kordali et al., 2005; Benli et al., 2007; Bloomer et al., 2011; Obolskiy et al., 2011; Raeisi et al., 2012; Reza et al., 2015; Abdollahnejad et al., 2016; Abtahi Froushani et al., 2016; Eidi et al., 2016; Tajbakhsh and Soleimani, 2018; Safari et al., 2019; Tajner-Czopek et al., 2020; Xu et al., 2020). Ethanol extracts of tarragon leaves reduced formalin and adrenalin edemas in rats up to 80% and were shown to have a potent anti-inflammatory action, stronger than that of phenylbutazone (Obolskiy et al., 2011). *A. dracunculus* aqueous extract (100 mg/kg) demonstrated a moderate effect on TNF-*α* and IL-6 (interleukin 6) release in fructose drinking water in male rats (Reza et al., 2015). The aqueous extract of tarragon (100 mg/kg for 21 consecutive days) significantly increased the level of anti-sheep red blood cells and caused a significant reduction in the production of pro-inflammatory IL-17 (interleukin 17) and IFN-*γ* (interferon *γ*) (Abtahi Froushani et al., 2016). The extract (50 and 100 mg/kg) showed significant activity in the xylene ear edema test in mice (Eidi et al., 2016).

## Conclusion

In the present study, the chemical composition of *Artemisia dracunculus* aerial part infusion was elucidated. This common spice is a source of compounds from the groups of phenolic acid derivatives and flavonoids. The potential anti-inflammatory activity of tarragon was evaluated in the human neutrophils model for the first time. The water extract obtained from *Artemisia dracunculus* herb was able to inhibit ROS, IL-8, and TNF-α production by PMNs in the imitated inflammation caused by neutrophil-stimulating bacterial factors such as f-MLP or LPS. Our results provide the background for the hypothesis that ADI, which is non-toxic for PMNs, exerts two direct activities needed in the case of bacterial infections. The first one is the resolution of bacteria-derived inflammation through the inhibition of neutrophils’ functions, which are the first line of immune defense. The second one is the direct activity against pathogens. ADI was characterized by moderate antibacterial activity against selected pathogenic Gram-negative and Gram-positive bacteria, including several *Staphylococcus aureus* and *Staphylococcus epidermidis* strains, which are the most common skin pathogens. *Staphylococcus* and their resistance to antibacterial drugs are one of the most important problems in hospital infections. The obtained results indicate the inhibitory effect of water *A. dracunculus* extract on *Corynebacterium diphtheria* and *Helicobacter pylori*, bacteria causing diphtheria and stomach ulcers, respectively. The investigated infusion from tarragon herb demonstrated a therapeutic potential to some extent, showing both the inhibition of cytokines’ secretion and antibacterial properties. Obtained results partially support the traditional usage of *Artemisia dracunculus* herb in the treatment of symptoms of bacterial infections-related inflammatory disorders. We believe that these properties may be used in the treatment of respiratory tract and skin infections. However, as flavonoids and phenolic acids usually undergo metabolic changes when administered orally, further research is needed and *in vivo* experiments are required to verify the extent of their systemic and topical activity.

## Data Availability Statement

The data are available on request to the corresponding author.

## Author Contributions

Investigation, Writing-Original Draft Preparation, Writing-Review and Editing: MM. Investigations: RH, SG, MC, AK, EO (plant material collection). Methodology, Review, and Editing: AK, MC, SG, RH.

## Funding

This project was carried out with the use of CePT infrastructure financed by the European Union’s European Regional Development Fund within the Operational Program “Innovative economy” for 2007–2013. The access publication fees were financially supported by Medical University of Warsaw.

## Conflict of Interest

The authors declare that the research was conducted in the absence of any commercial or financial relationships that could be construed as a potential conflict of interest.
